# Identification of New, Translatable ProtectomiRs against Myocardial Ischemia/Reperfusion Injury and Oxidative Stress: The Role of MMP/Biglycan Signaling Pathways

**DOI:** 10.3390/antiox13060674

**Published:** 2024-05-30

**Authors:** Tamara Szabados, Arnold Molnár, Éva Kenyeres, Kamilla Gömöri, Judit Pipis, Bence Pósa, András Makkos, Bence Ágg, Zoltán Giricz, Péter Ferdinandy, Anikó Görbe, Péter Bencsik

**Affiliations:** 1 Cardiovascular Research Group, Department of Pharmacology and Pharmacotherapy, Albert Szent-Györgyi Medical School, University of Szeged, Dóm tér 12, H-6720 Szeged, Hungary; szabados.tamara@med.u-szeged.hu (T.S.); arnold.molnar@pharmahungary.com (A.M.); evakenyeres85@gmail.com (É.K.); kamilla.gomori@gmail.com (K.G.); pipis.judit@med.u-szeged.hu (J.P.); posa.bence78@gmail.com (B.P.); gorbe.aniko@semmelweis.hu (A.G.); 2 Pharmahungary Group, Hajnóczy u. 6, H-6722 Szeged, Hungary; bence.agg@pharmahungary.com (B.Á.); giricz.zoltan@semmelweis.hu (Z.G.); peter.ferdinandy@pharmahungary.com (P.F.); 3 Cardiometabolic and HUN-REN-SU System Pharmacology Research Group, Department of Pharmacology and Pharmacotherapy, Faculty of Medicine, Semmelweis University, Nagyvárad tér 4, H-1089 Budapest, Hungary; makkos.andras@semmelweis.hu

**Keywords:** biglycan, ischemic conditioning, matrix metalloproteinase, microRNA, myocardial ischemia/reperfusion injury, pig, porcine, protectomiR

## Abstract

Introduction: Ischemic conditionings (ICon) were intensively investigated and several protective signaling pathways were identified. Previously, we have shown the role of matrix metalloproteinases (MMP) in myocardial ischemia/reperfusion injury (MIRI) and the cardioprotective role of biglycan (BGN), a small leucine-rich proteoglycan in vitro. Here, we hypothesized that cardiac MMP and BGN signaling are involved in the protective effects of ICon. Methods: A reverse target-microRNA prediction was performed by using the miRNAtarget™ 2.0 software to identify human microRNAs with a possible regulatory effect on MMP and BGN, such as on related genes. To validate the identified 1289 miRNAs in the predicted network, we compared them to two cardioprotective miRNA omics datasets derived from pig and rat models of MIRI in the presence of ICons. Results: Among the experimentally measured miRNAs, we found 100% sequence identity to human predicted regulatory miRNAs in the case of 37 porcine and 24 rat miRNAs. Upon further analysis, 42 miRNAs were identified as MIRI-associated miRNAs, from which 24 miRNAs were counter-regulated due to ICons. Conclusions: Our findings highlight 24 miRNAs that potentially regulate cardioprotective therapeutic targets associated with MMPs and BGN in a highly translatable porcine model of acute myocardial infarction.

## 1. Introduction

Despite detailed guidelines for the management of ST-elevation myocardial infarction (STEMI) having been available for decades, acute myocardial infarction (AMI) remains a major cause of mortality and morbidity in developed countries [1]. While ischemic conditioning (ICon) techniques have demonstrated high potency for cardioprotection in preclinical studies, the translation of these findings to clinical settings for meaningful cardioprotection has proven challenging except for ischemic preconditioning, which has limited clinical relevance. Therefore, it is crucial to identify the mechanisms involved in animal models to successfully translate cardioprotective efficacy of ICons to humans. 

It is well known that increased oxidative stress during myocardial ischemia reperfusion injury (MIRI) triggers the activation of matrix metalloproteinase-2 (MMP-2) contributing to myocardial damage [2]. MMP-2 is abundant in the heart, expressed by various cell types including cardiomyocytes, endothelial cells, vascular smooth muscle cells, macrophages, and fibroblasts (see for review [3]), and becomes highly activated in response to oxidative stress [4]. MMP-2 targets various components of the extracellular matrix, such as laminin, elastin, type IV collagen, and fibronectin, as well as intracellular proteins like troponin I and myosin light chain 1, thereby leading to contractile dysfunction [5,6,7]. MMP-2 and MMP-9 are released rapidly following MIRI thereby resulting in local tissue injury [8]. Upon entering the circulation, they can also damage remote/distant organs [9]. In vitro studies indicate an elevation in MMP-9 activity produced by cardiac fibroblasts in response to hypoxia and oxidative stress [10,11]. There has been abundant evidence that the MMP-9 level is elevated in the plasma and in the left ventricle after MI in preclinical and clinical studies as well [12,13]. Although, MMP-9 activity can be detected in vivo from cardiac fibroblasts of ventricular samples, the vast majority of its source derives from blood cells such as macrophages and monocytes but not cardiac myocytes that do not express MMP-9 (Iyer et al. [14]). 

We have previously shown that biglycan, a prominent member of small leucine-rich proteoglycans, can exert cytoprotection on myocardial cells against simulated IRI when administered exogenously. This cardioprotective effect was accompanied by a significant increase in the nitric oxide (NO) production of cardiomyocytes [15]. Beside the NO-dependent mechanism of the cytoprotection, we showed that toll-like receptor-4-mediated mechanisms, such as activation of ERK, JNK, and p38 MAP kinases, are also involved in the cardio-cytoprotective effects of biglycan [16]. Previous in vitro studies demonstrated that biglycan can be served as a substrate for either MMP-2 or MMP-9 [17,18]. According to the best of our knowledge, this is the first study to evaluate the changes of cardiac and plasma biglycan levels in myocardial ischemic pre-, post-, or remote ischemic perconditionings.

In previous preclinical studies, our research group showed that ICons accompany significant cardioprotective effects both in rodents as well as in large animals. Ischemic pre- and postconditioning reduced infarct size in an ex vivo rat model of AMI [19]. In a clinically highly relevant closed-chest porcine model of AMI, ischemic preconditioning significantly decreased myocardial infarct size, while ischemic post- and remote perconditionings provided vasculoprotective and cardioprotective effects by reducing myocardial microvascular obstruction and cardiac edema [20]. In the above studies, analysis of the myocardial microRNA expression pattern by bioinformatics revealed significant changes after different conditionings in both rat and porcine models, revealing potential underlying mechanisms for cardioprotection.

MicroRNAs (miRNA) are short, approximately 18–25 nucleotide-long non-coding RNA sequences that are already available as advanced therapy medicinal products (extensively reviewed elsewhere [21]). MiRNAs negatively regulate gene expression at the post-transcriptional level either by inhibiting the translation or promotion of degradation of the target mRNAs [22,23]. Complex cellular pathways can be regulated by a single miRNA [24], and due to their pleiotropic nature, they gain great potential to become multi-target drugs for diseases with multifactorial origin [25].

Therefore, in this study, the aim was to identify potential novel therapeutic target miRNAs that regulate MMP- and BGN-mediated signaling pathways of ICons to enhance the translation of the cardioprotective effect of ICons into effective therapeutic strategies for human patients undergoing cardiovascular events. Here, we hypothesized that miRNAs influencing cardiac MMP and BGN signaling contribute to the protective effects of ICons and investigated their expression pattern measured in rats as well as in a highly translatable porcine model of MIRI in the presence of ICons. Furthermore, sequence similarity analysis was used to assess only miRNAs from both species that testified 100% sequence identity with human miRNA sequences, and miRNAs, which play a major role in ICons, have also been shown to play a role in cytoprotection against oxidative stress.

## 2. Materials and Methods

### 2.1. In Vivo Closed-Chest Porcine Model of Acute Myocardial Infarction

**Figure 1:**
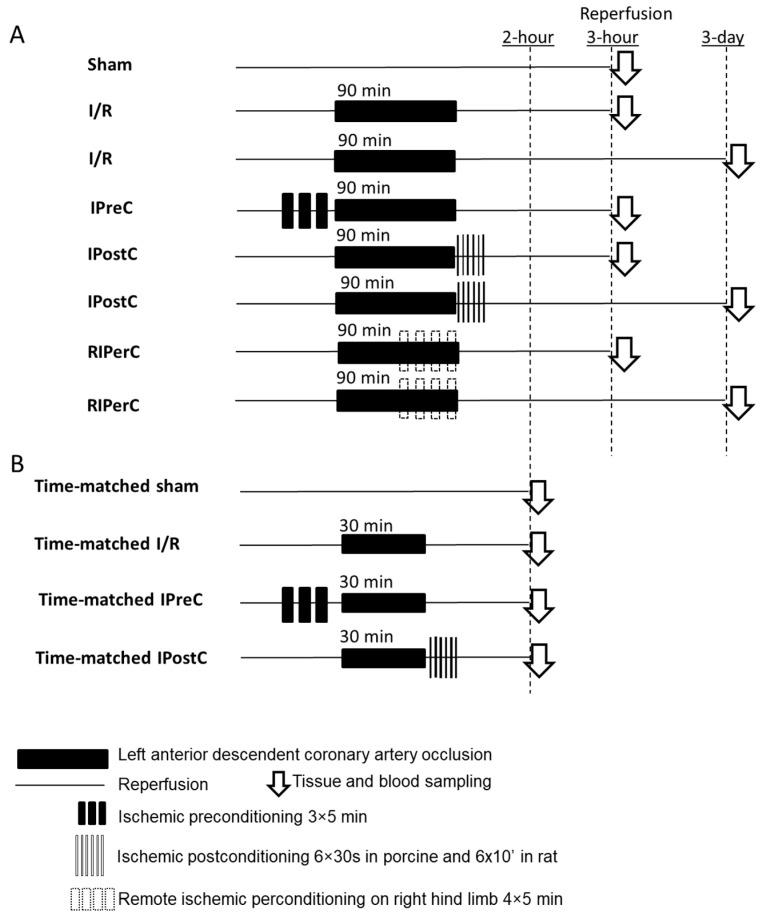
Experimental protocols for animal models of microRNA measurements. Panel (**A**): Protocols for ischemic conditionings in a clinically relevant closed-chest porcine model of reperfused acute myocardial infarction (for original protocol figure, see Baranyai et al. [20]). Panel (**B**): Protocols for ischemic conditionings in rat model of reperfused acute myocardial infarction (for original protocol figure, see Varga et al. [19]).

The detailed experimental protocol has been published previously by Baranyai et al. [20]. The modified experimental protocol figure is shown in Figure 1, Panel A. Acute myocardial infarction (AMI) was induced in female domestic pigs by balloon catheter placed in the mid part of the left anterior descending coronary artery. For induction of AMI, the intracoronary balloon was inflated with 5 atm for 90 min, following either 3 h or 3 days of reperfusion by deflation of the balloon. Porcines were randomized into 5 surgical groups: sham, ischemia only, ischemic preconditioning (IPreC), ischemic postconditioning (IPostC), and remote ischemic perconditioning (RIPERC). In the IPreC group, LAD was occluded by the inflation of the balloon 3 times at 5 atm for 5 min followed by 5 min of reperfusion prior to 90 min ischemia. In the IPostC group, animals were given six cycles of 30 s occlusion/reperfusion of the LAD after the 90 min ischemia. In the RIPERC group, 4 cycles of 5 min occlusion and 5 min reperfusion of the femoral vessels were performed by the tightening and releasing of a snare around the right hind limb, starting at the 50th min of LAD occlusion.

### 2.2. Ex Vivo Rat Model of Acute Myocardial Infarction 

The detailed experimental protocol was published previously by Varga et al. [19]. Briefly, hearts were isolated from male Wistar rats and subjected to time-matched sham operation or 30 min ischemia and 120 min reperfusion or preconditioning (3 × 5 min of coronary occlusion) followed by ischemia–reperfusion or ischemia–reperfusion followed by postconditioning (6 × 10 s of global ischemia–reperfusion at the onset of reperfusion). The modified experimental protocol figure is shown in Figure 1, Panel B.

### 2.3. Tissue and Plasma Collection from Porcine Model

Blood and tissue samples were collected at termination of the animals (either 3 h or 3 days). Blood samples were centrifuged at 2000× *g* for 10 min and stored at −80 °C until the analysis was performed. Hearts were removed and placed in ice-cold saline. Infarcted left ventricular tissue was isolated from the distal anterior infarcted area (below the origin of the second diagonal branch, appearing as macroscopic hemorrhage with edema at three hours or pale-grey color at three days), and in the case of sham operation, tissue samples were isolated from an equivalent location. Samples were snap-frozen immediately and stored at −80 °C until analysis. For the original description, see Baranyai et al. [20].

### 2.4. MicroRNA Measurement with High-Throughput qRT-PCR from Porcine Infarcted Cardiac Tissue

MicroRNAs were measured from infarcted left ventricular tissue at 3 h of reperfusion using a High Pure miRNA Isolation Kit (Merck KGaA, Darmstadt, Germany) according to the manufacturer’s instructions. The quantity of miRNA was measured by a NanoDrop 1000 spectrophotometer (Thermo Fischer Scientific, Waltham, MA, USA; RRID:SCR_016517), and miRNA was converted into cDNA with a MicroRNA Reverse Transcription Kit (Thermo Fisher Scientific; Cat# 4366597). For the measurement of the specific microRNA expression pattern in the pig myocardium, 221 pig miRNAs were selected based on the miRBase Release 20 (June 2013), that were further confirmed to be expressed in pig myocardium with a literature search in PubMed until 2016 January. Real-time PCR was performed, and curves were analyzed by using dynamic tube and slope correction methods. Results were calculated with the ΔΔCp evaluation method. 

### 2.5. MicroRNA Measurement with Microarray Analysis from Rat Infarcted Cardiac Tissue 

MicroRNAs were measured from the infarcted anterior wall of the left ventricle, and from an equivalent location without ischemia in the case of sham operation, at the end of the experimental protocol using an miRNA Complete Labeling and Hyb Kit system (Agilent Technologies, Palo Alto, CA, USA) and were validated with quantitative real-time PCR. For a detailed description, see Varga et al. [19].

### 2.6. ELISA Assay to Determine Biglycan Level in Cardiac Porcine Tissue and Plasma Samples

An ELISA assay to determine biglycan level was performed and analyzed by a double antibody sandwich technique ELISA kit (MBS2602077 MyBiosource, San Diego, CA, USA) according to the manufacturer’s instructions. Each sample was measured in duplicate; the average values are used as final data. The concentration was calculated according to standard concentrations, and the corresponding optical density was measured at 450 nm by an Optima FluoStar plate reader (BMG Labtech, Ortenberg, Germany).

### 2.7. Zymography Assay to Measure MMPs in Cardiac Porcine Tissue and Plasma Samples 

To assess MMP activities from porcine tissue and plasma samples, gelatin zymography was performed as previously described in detail [26]. Briefly, 8% SDS-polyacrylamide were copolymerized with 2 mg/mL gelatin (Sigma-Aldrich, type A from porcine skin), and 50 μg of total protein (measured by BCA kit; 23225, Thermo Fischer Scientific, Waltham, MA, USA) per lane was loaded and separated by electrophoresis (90 V, 90 min). After electrophoresis, gels were washed with renaturation buffer (Novex, Carlsbad, CA, USA) for 40 min and then incubated for 40 h in the case of tissue homogenate and 20 h in the case of plasma samples at 37 °C in zymography development buffer (Novex, Carlsbad, CA, USA). Gels were stained with a 0.05% Coomassie Brilliant Blue (Sigma-Aldrich, St. Louis, MO, USA). MMP activity was detected as transparent bands against a blue background. A mixture of recombinant, human MMP-2 and MMP-9 was used as positive control. Band intensities were quantified by densitometry using Quantity One software (version 4.6.9; Bio-Rad, Hercules, CA, USA), and values were expressed in arbitrary units. A representative gel image is provided in the Supplementary Materials (Supplementary Figure S1). MMP-9 activity from left ventricular samples was not determined to avoid misinterpretation of the data. Although MMP-9 activity can be detected from ventricular samples, its source are cardiac fibroblasts and mainly blood cells such as macrophages and monocytes but not cardiac myocytes, which do not express MMP-9. Furthermore, porcine hearts could not be decontaminated from blood, and thereby the source of MMP-9 activity would be equivocal and misleading since the amount of ventricular remnant blood can be variable between samples.

### 2.8. Network Theoretical Reverse Target-microRNA Prediction

To identify human miRNAs that can directly or indirectly affect the expression and activity of MMP-2/9 and biglycan, two separate network theoretical reverse target-microRNA predictions were performed. The input gene list for these two predictions was constructed based on a literature search by collecting genes involved in the regulation of MMP-2/9 and biglycan, respectively. Medical subject headings (MeSH) terms/search strings were used to find appropriate signaling molecules related to MMP-2/9 and biglycan. We used “matrix metalloproteinase-2” or -9 as well as “biglycan” in combination with “activation” or “inhibition” or “negative regulator”. We selected review articles from the hits that resulted from the abovementioned literature search, and the collected proteins were assigned as regulatory factors for MMPs or BGN. For MMP-2/9, as a second step, the original literature-search-based gene list was extended by transcription factors that according to the TRRUST database [27] regulate the expression of genes in the original gene list (Supplementary Table S1). MicroRNA–target interaction networks were constructed by the reverse target-microRNA prediction mode of the miRNAtarget™ (Pharmahungary, Szeged, Hungary) software. To ensure high accuracy, in contrast to a previous reverse [28] and several forward prediction studies [29,30,31], in this case only the experimentally validated miRTarBase database (v7.0) [32] was used as a microRNA–target interaction data source. The constructed miRNA–target interaction networks are bipartite graphs. In a bipartite graph, nodes are divided into two sets, and all edges connect a node from one of the sets to a node belonging to the other set (i.e., no edges are allowed between nodes of the same set) (Pavlopoulos et al. [33]). In the case of miRNA–target interaction networks, the two sets of nodes are genes and human miRNAs predicted to regulate these genes, and all edges represent predicted miRNA–target interactions (i.e., there are no edges between two gene nodes or two miRNA nodes). The degree of a node is defined as the number of edges incident to the node. Considering the bipartite characteristic of miRNA–target interaction networks, the degree of an miRNA node is the number of edges connecting the miRNA node to gene nodes, or in other words, it is the number of target genes predicted to be regulated by the miRNA. Therefore, in this study, the degree of a miRNA node measures to what extent the miRNA is expected to be involved in the regulation of the MMP-2/9 and/or biglycan signaling pathways. For visualization of the predicted microRNA–target interaction networks, the EntOptLayout plugin (version 2.1) [34] for the Cystoscape network analysis framework (version 3.9.1) [35] was used (Supplementary Figures S2 and S3). Homologous porcine and rat miRNAs for the human miRNAs were selected based on 100% sequence identity using mature miRNA sequence data of the miRBase (version 22.1) database [36].

### 2.9. Literature Search and Database for Oxidative Stress/Antioxidant Protection-Related miRNAs 

We have performed a thorough literature search with the miRNAs exhibiting significant counter-regulation after MIRI in the absence or in the presence of different ICons to reveal the relationship of miRNAs with potential cardioprotective effects either to oxidative stress and/or to antioxidant protection. Therefore, we used the following search strings in the PubMed database—“(heart OR myocard*) AND (ischem* OR reper* OR infarct*) AND (miRNA OR microRNA OR “non-coding RNA” OR “non coding RNA”) AND (“oxidative stress” OR “nitrative stress” OR antioxidant OR “free radical”)”—to filter all miRNAs involved both in oxidative/antioxidant signalization as well as in MIRI. We found 453 articles according to the above keywords. Then, results were exported to csv files and converted to MS Excel sheets, where further filtering for each individual miRNA was conducted. Moreover, to find all the available publications related to oxidative stress or antioxidant protection, the following search string was used in PubMed for each of the counter-regulated miRNAs: “(miR-NN OR microRNA-NN) AND (“oxidative stress” OR “nitrative stress” OR “nitrosative stress” OR antioxidant OR “free radical)” where NN is for the number of the particular miRNA. The resulting hits were exported to csv and converted to MS Excel files to create a database, which then was filtered for heart-related publications.

### 2.10. Statistical Analysis

Data from MMP and BGN are expressed as mean ± sem. data are compared to the ischemic control group using one-way ANOVA with Dunnett’s multiple comparisons test, *n* = 4–8/Group; *p* < 0.05 was considered significant. In the sample size of ventricular and plasma samples, minor discrepancies can be observed due to excluded values based on outlier analysis, which was performed to exclude values outside the mean ± 2 SD. For visual comparison and representation of both down- and upregulation changes in miRNA expression, data were shown as log2 changes. Averages of log2 changes were plotted with SD values.

## 3. Results

### 3.1. Selection of MMP/BGN-Related Genes Based on Literature Search and Construction of Predicted Regulatory microRNA–Target Interaction Networks

**Figure 2:**
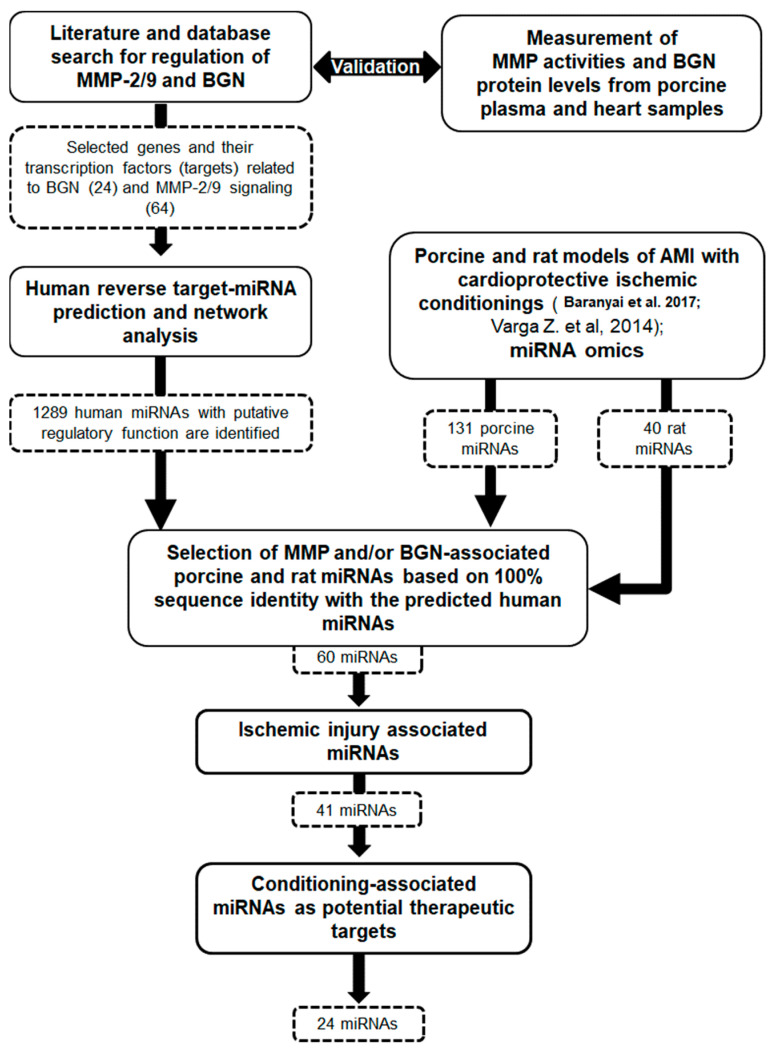
Study design with results [19,20].

In order to validate the role of miRNAs targeting MMP-2 and -9 enzymes and biglycan in acute myocardial infarction and in different types of conditioning, we have built two reverse prediction-based human miRNA–target interaction networks using genes involved in the regulation of MMPs and BGN as input. As a result of a thorough literature search and querying the TRRUST database for the transcription factors related to the MMP pathway, including the own genes of BGN and the two MMP isoforms and their regulatory factors, we have identified 24 and 64 different signaling molecules for the BGN and the MMP pathways, respectively (Supplementary Table S1). The constructed miRNA–target interaction networks contain 549 miRNAs related to BGN regulation and 1137 miRNAs related to MMP-2 and -9 regulation (Supplementary Tables S2 and S3 and Supplementary Figures S2 and S3). Since there are overlapping miRNAs, the networks ultimately consist altogether of 1289 miRNAs (see flow chart in Figure 2).

### 3.2. Effect of Ischemic Conditionings on Porcine Cardiac miRNAs

MicroRNAs were isolated and measured from infarcted left ventricular tissue samples from our previous closed-chest porcine model of AMI at 3 h of reperfusion. Out of the 220 microRNAs measured from porcine left ventricles, 103 showed a significantly altered expression during one or more of the ischemic conditionings as compared to the ischemic control. Beyond these 103 miRNAs, we found 28 further miRNAs, which showed a significantly altered expression in the ischemic control group as compared to the sham-operated animals but not between the ischemic control and ischemic conditionings. The above 131 miRNAs altogether were subjected to further analyses investigating their role in ischemic injury and/or ischemic conditionings.

### 3.3. Effect of Ischemic Conditionings on Rat Cardiac miRNAs

MicroRNAs were isolated from left ventricular samples derived from isolated rat hearts subjected to 30 min ischemia and 2 h of reperfusion. A total of 350 cardiac miRNAs were evaluated in the rat, and 40 exhibited a significantly altered expression as compared to the ischemic control group.

### 3.4. Interspecies Sequence Similarity Analysis

The sequences of the differentially expressed rat and porcine miRNAs were compared to the predicted human miRNAs. In the case of the porcine model, from the 131 differentially expressed miRNAs, 37 (Table 1), and from the 40 differentially expressed rat miRNAs, 24 miRNAs (Table 2) showed 100% sequence identity with the predicted human miRNA sequences. All the above miRNAs were related either to MMPs and/or to BGN.

These miRNAs have been further analyzed, and among the 60 miRNAs (one miRNA was overlapping between the two species), 41 (one miRNA was overlapping between the two species) were identified as being associated with ischemic injury, of which twenty-four (sixteen from porcine and eight from rat; one miRNA was overlapping between the two species) were counter-regulated due to at least one type of ischemic conditionings (Table 3). These miRNAs demonstrate potential cardioprotective effects; therefore, according to our previously published data (see [19] for details), we identified them as “protectomiRs”.

### 3.5. Ischemic Injury-Associated miRNAs

Forty-one miRNAs (25 out of 37 porcine + 17 out of 24 rat miRNAs; one miRNA was overlapping between the two species) were found to be associated with ischemic injury by significantly altering their expression measured in the early phase of reperfusion (i.e., 2 or 3 h after its onset in rat and porcine models, respectively) after myocardial ischemia as compared to the sham-operated animals. One miRNA was associated with BGN signaling only (miR-652-3p, downregulated), and 16 miRNAs were related to MMPs only. Ischemic injury caused upregulation in cases of miR-369-3p, miR-196a-5p, miR-23a-3p, miR-126-3p, miR-101-3p, miR-193a-5p, and miR-338-3p, while ischemic injury caused downregulation in cases of miR-320c, miR-7-5p, miR-215-5p, miR-450a-5p, and miR-135a-5p in the porcine model and miR-320a, miR-331-3p, miR-378a-5p, and miR-877-5p in the rat model.

The remaining 25 miRNAs were related to both MMPs and BGN signaling pathways. Ischemia caused upregulation in the case of four porcine miRNAs—miR-34a-5p, miR-128-3p, miR-9-5p, and miR-193a-3p—and in the case of two rat miRNAs—miR-19b-3p, miR-19a-3p—while ischemia caused downregulation in case of nine porcine miRNAs—miR-106b-5p, miR-18b-5p, miR-30b-5p, miR-361-5p, miR-107, miR-26a-5p, let-7a-5p, miR-19b-3p, and 425-5p—and in case of nine rat miRNAs—miR-33a-5p, let-7b-5p, miR-335-5p, let-7c-5p, let-7a-5p, miR-125a-5p, miR-181a-5p, miR-218-5p, let-7d-5p, and let-7f-5p (see Table 1 and Table 2 for details).

### 3.6. Cardioprotection-Associated miRNAs

Altogether, we identified 24 protectomiRs (i.e., conditioning-associated miRNAs) that showed significantly altered expression in ischemic control as compared to the sham-operated animals and were simultaneously significantly counter-regulated by any of the ischemic conditionings (i.e., IPreC, IPostC, and/or RIPerC vs. ischemic control). Three miRNAs were found to be counter-regulated by all three ischemic conditionings as compared to the ischemic control: miR-34a-5p, miR-193a-3p (related to the biglycan and MMP regulatory pathway), and miR-450a-5p (related to MMP pathway).

Two miRNAs, miR-361-5p and miR-320a, were both counter-regulated by IPreC and IPostC as well, which are related to MMP, BNG, and MMP regulatory pathways, respectively. Four miRNAs showed significant counter-regulation by IPostC and RIPerC—miR-193a-5p, miR-23a-3p, miR-196a-5p, and miR-369-3p—which are related only to the MMP pathway. Similarly, IPreC along with RIPerC showed a significant counter-regulation as compared to the ischemic control in mir-26a-5p and miR107 expression, which are related to both the BGN and MMP pathways. 

Several other miRNAs, including miR-338-3p; let-7 family members, such as let-7a-5p, let-7b-5p, let-7c-5p, let-7d-5p, and let-7f-5p; miR-181a-5p; and miR-335-5p, were counter-regulated by IPostC only, while RIPerC alone showed a counter-regulation in the expression of miR-9-5p, miR-128-3p, miR-215-5p, and miR-425-5p. MicroRNA miR-128-3p was the most prominently downregulated miRNA, effected only by RIPerC, which is related to both MMP and BGN signaling pathways (see Table 3 for details).

### 3.7. Effect of Ischemic Conditionings on Matrix Metalloproteinases and Biglycan in the Porcine Model

**Figure 3:**
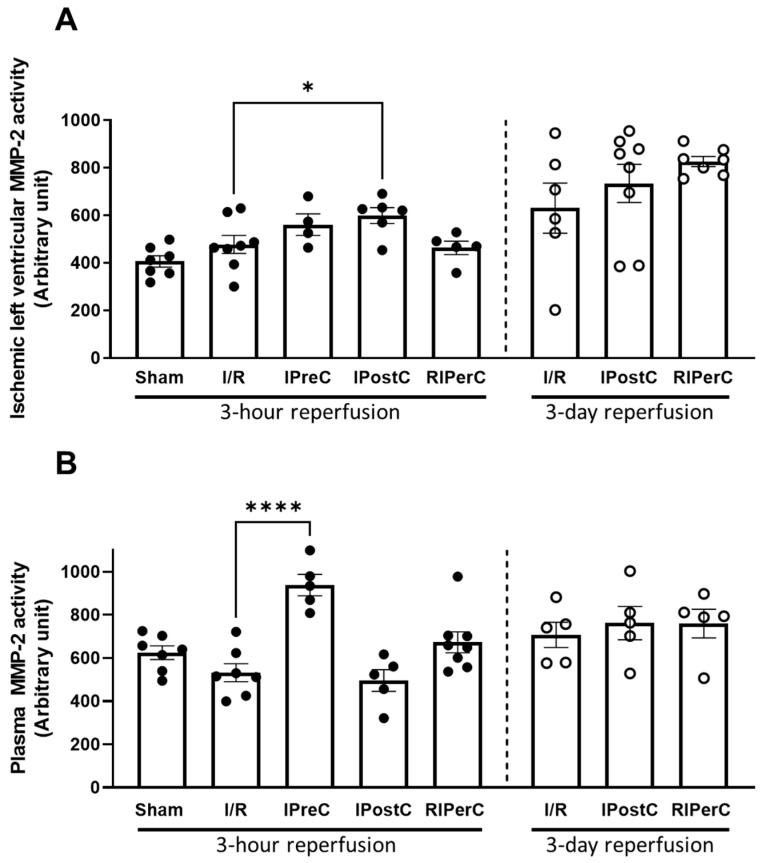
Matrix metalloproteinase-2 activity measured from infarcted left ventricular tissue (Panel (**A**)) and plasma samples (Panel (**B**)) of pigs subjected to acute myocardial infarction and ischemic conditioning interventions. Results are expressed as mean ± SEM. Statistical analysis shows the results of one-way ANOVA with Dunnett’s multiple comparisons test with the corresponding control; *n* = 4–8/group, * *p* = 0.0468, **** *p* < 0.0001.

We measured the matrix-metalloproteinase-2 and -9 activities with gelatine zymography from porcine plasma and the MMP-2 activity from porcine left ventricular ischemic tissue at 3 h and 3 days of reperfusion following the different ischemic conditionings (Figure 3). Three hours after the onset of reperfusion, cardiac MMP-2 activity was significantly increased after IPostC (Figure 3, Panel A), while plasma MMP-2 activity was significantly increased after IPreC (Figure 3, Panel B). Three days after the onset of reperfusion none of the conditionings showed a significant impact on MMP-2 activity as compared to the corresponding ischemic control either in the left ventricle or in the plasma. 

**Figure 4:**
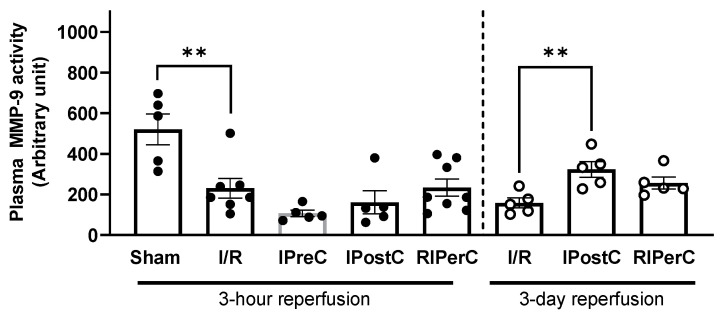
Matrix metalloproteinase-9 activity measured from plasma samples of pigs subjected to acute myocardial infarction and ischemic conditioning interventions. Results are expressed as mean ± SEM. Statistical analysis shows the results of one-way ANOVA with Dunnett’s multiple comparisons test with the corresponding control; *n* = 5–8/group, ** *p* < 0.01.

Plasma MMP-9 activity was significantly decreased in ischemic control as compared to the sham-operated animals, while ischemic conditionings did not affect MMP-9 activity at early reperfusion (i.e., 3 h); however, at 3-day of reperfusion, MMP-9 activity was significantly increased after IPostC as compared to the 3-day ischemic control samples (Figure 4).

**Figure 5:**
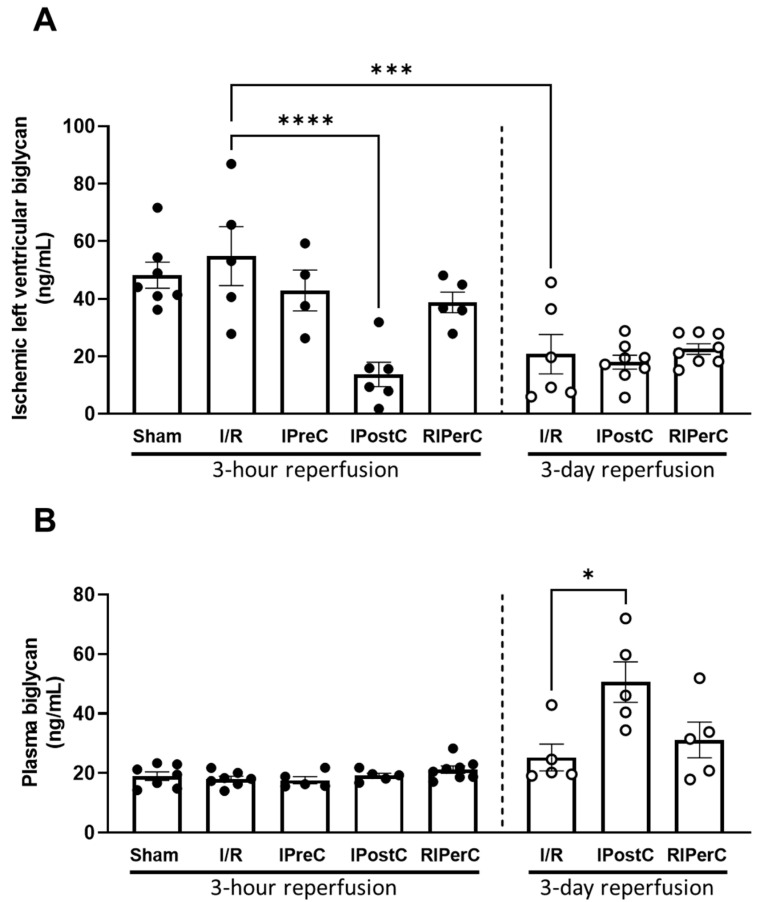
Biglycan concentration measured from infarcted left ventricular tissue (Panel (**A**)) and plasma samples (Panel (**B**)) of pigs subjected to acute myocardial infarction and ischemic conditioning interventions. Results are expressed as mean ± SEM. Statistical analysis shows the results of one-way ANOVA with Dunnett’s multiple comparisons test with the corresponding control; *n* = 4–8/group, * *p* = 0.0184, *** *p* = 0.0003, **** *p* < 0.0001.

Biglycan concentration was measured by using ELISA technique. In the ischemic left ventricle, the biglycan level was significantly reduced in the IPostC group either 3 h or 3 days after the onset of reperfusion as compared to the 3 h ischemic control; however, this difference was lost at 3 days of reperfusion due to a significant decline in BGN levels in the control ischemic group (Figure 5, Panel A). In plasma samples, at 3 h of reperfusion, no difference was found between the groups, while at 3 days of reperfusion, the plasma biglycan level was significantly elevated after IPostC (Figure 5, Panel B). Since biglycan is a substrate of MMP-2, the increase in cardiac MMP-2 activity in the IPostC group at 3 h of reperfusion may be related to a decrease in cardiac biglycan concentration.

### 3.8. Conditionings-Related miRNAs’ Role in the Cardiac Oxidative Stress/Antioxidant Processes

From the available literature related to the above miRNAs as well as to oxidative stress and cardiac pathologies, we have selected papers published in journals with the highest ranking (Q1 or D1 if available). We summarized the most important and most relevant findings separately, which are simultaneously related to cardiac and oxidative pathologies (Table 4).

## 4. Discussion

This is the first demonstration in the literature of early and late changes of circulatory as well as ischemic myocardial MMP-2, -9, and biglycan levels after different types of Icons, including IPreC, IPostC, and RIPerC in a rat, and clinically relevant porcine models of MIRI. Furthermore, after thorough sequence similarity analyses, we have identified myocardial miRNAs that are involved in the signaling processes of the above proteins and are simultaneously counter-regulated after ischemic injury and in the presence of one or more ICons. Moreover, we further added roles in oxidative stress and/or in antioxidant signaling pathways of the identified and counter-regulated miRNAs above. Thereby, via a complex approach based on experimental measurements, on available databases, as well as on bioinformatic analysis, here we have provided valuable knowledge on the network of myocardial miRNAs and either MMP–biglycan or oxidative stress/antioxidant signaling pathways for future investigations and for potential drug development against MIRI.

### 4.1. Cardioprotection by MMP- and BGN-Related Reduction of Myocardial Edema and Microvascular Obstruction

Previously, we have shown that IPreC, IPostC, and RIPerC can protect the heart against microvascular damage in MIRI by reducing myocardial edema and microvascular obstruction (MVO) in a clinically relevant closed-chest porcine model of ischemia reperfusion injury [20]. 

The formation of coronary microvascular dysfunction and obstruction related to MIRI is well-established, as well as the correlation between the microvascular dysfunction and the increased risk for cardiovascular events (see for review [51]). In an acute rat model of ischemia/reperfusion we have shown that although IPreC and two newly developed MMP-2 inhibitors decreased infarct size significantly, only IPreC was able to reduce MVO parallelly [52]. Cardioprotection by inhibition of MMP-2, MMP-9, and MMP-14 via intramyocardial injection of hyaluronic acid hydrogel-releasing TIMP-3 was shown to reduce infarct size in Yorkshire pigs 10–14 days post MI [53]. Very recently, it was shown that intracoronary hypothermia resulted in improved ejection fraction and reduced MVO as well as reduced MMP-9 level in a porcine model of MIRI [54]. The link between MMPs and the formation of microvascular obstruction is unclear, but we believe that the regulation of MMPs may play a significant role in that process.

Edema is known to significantly contribute to reduced cardiac performance. Cell swelling was shown to be directly associated with decreased contractility in isolated myocytes exposed to osmotic stress [55].

In a porcine model of myocardial infarction with IPreC, Ramirez-Carracedo et al. showed that inhibiting the extracellular matrix metalloprotease inducer (EMMPRIN) reduced infarct size, microvascular obstruction, intramyocardial hemorrhage, and edema and improved cardiac performance as seen by left ventricle ejection fraction 7 days post MI [56]. After a 45 min coronary occlusion, gadolinium-containing nanoparticles conjugated with AP9 (NAP9) targeting EMMPRIN and a control nanoparticle was injected. The targeted EMMPRIN plays a key role in regulating the expression of many matrix metalloproteinases, among others those of MMP-2 and MMP-9 [57]. The authors suggested that NAP9 could prevent induction of MMPs through inhibiting the EMMPRIN, and thus improving cardiac function post MI [56].

In a Langendorff-perfused isolated rat heart model of ischemia/reperfusion injury, Fert-Bober and colleagues studied the effect of MMP-2 inhibitors (o-phenanthroline or doxycycline). The inhibitors reduced the release of endothelial integrity markers, interstitial albumin, and serotransferrin and abolished the edema formation observed after histological analysis, evidenced by a decrease in extracellular space and in the number of vacuolated myocytes, as well as by intact endothelial linings of the capillaries and larger blood vessels [58].

The literature about the relation of biglycan and edema is very limited; only four hits were found when searching in PubMed with the search string biglycan AND (edema or oedema). A few articles showing the results of biglycan measured from corneal edema, from fetuses with Turner syndrome, bronchial vascular remodeling in asthma, and dexamethasone-treated rats were found. There is no information about the relation of biglycan and edema with cardiac origin. Similarly, literature search on the association of biglycan and microvascular obstruction or dysfunction did not yield any relevant result. 

### 4.2. MiRNAs Related to MMP/Biglycan Signaling Are Included in Oxidative Stress/Antioxidant Protection

According to the available literature, miRNAs associated with the signaling processes of MMPs and biglycan are not only involved in the cardioprotective effects of ICons, but they are also related to oxidative damage of cardiac myocytes as we demonstrated in Table 4. Here, we focus on miRNAs displaying significant counter-regulation after ischemic injury and a response to ICons with the literature data. The majority of counter-regulated miRNAs in the porcine model showed an increase in expression following MIRI and a decrease in expression following any of the conditioning treatments, while a minority of the miRNAs have oppositely changed, i.e., downregulated following MIRI and overexpressed following ICons. In the rat model, we found miRNAs only with downregulation after MIRI and overexpression following any of the ICons. Since we used miRNAs from both species that testified 100% sequence identity with human miRNA sequences, we performed the literature search according to the human miRNA nomenclature (see Table 4). In cases of miR-34a-5p, miR-23a-3p, miR-9-5p, miR26a-5p, miR-425-5p, miR-181a-5p, and miR-335-5p, we found consistent data on ischemic heart disease in the literature. However, interestingly, contradictory effects have been reported for the other miRNAs on either oxidative stress or on antioxidant protection that we found in our models. 

### 4.3. Mitochondrial-Related miRNAs, mitomiRs

A subset of microRNAs that regulate mitochondrial function, termed as “mitomiR”, has recently been the focus of many studies investigating mitochondrial dysfunction related to disease pathologies, such as mitochondrial homeostasis, which is paramount in myocardial infarction [59]. MiR-361 (mimic was suggested to be cardioprotective in the present study) was described as a mitochondrial fission promoter and initiated apoptosis in cardiomyocytes in vitro and in vivo as well through negatively regulating the prohibitin 1 protein located in the mitochondrial inner membrane and thus controlling the mitochondrial dynamics and network [59,60]. This finding suggests that antagomiR-361 could serve as a therapeutic tool in cardiac disease, which is contradictory to our findings. MiR-23a (antagomiR was suggested to be cardioprotective in the present study) was reported to exacerbate cardiomyocyte apoptosis and promote mitochondrial fission by targeting peroxisome proliferator-activated receptor gamma coactivator-1α, which is in line with our results [38].

Another ProtectomiR, miR-34a (antagomiR was suggested to be cardioprotective in the present study), was shown to be associated with the negative regulation of mitochondrial biogenesis and mitophagy (mitochondria-specific autophagy) by targeting sirtuin-1 [61]. MiR-181a (mimic was suggested to be cardioprotective in the present study) was also shown to be associated with the negative regulation of mitophagy by inhibiting the degradation of mitochondrial proteins by directly targeting Parkin E3 ubiquitin ligase [62]. However, the abovementioned two studies were not related to cardiovascular pathologies.

## 5. Limitations

The present work has some limitations. Tissue and plasma samples from porcine ischemic preconditioning group after 3 days of reperfusion were not available; therefore, we cannot provide any data from that group. A further limitation is that in the rat model—since Langendorff-perfused isolated hearts were used—the hearts could be subjected only to coronary occlusion and reperfusion with or without ischemic pre- or postconditioning but not remote ischemic perconditioning; therefore, we cannot provide any information about the miRNA expression pattern in that group. Furthermore, we have not measured MMP/biglycan levels as well as the presence of MVO and edema in rats. Thus, we have only limited availability of previous data on MMP, MVO, and edema regarding myocardial ischemia and ischemic preconditioning (see [52,63] for more details). Biglycan levels were never determined in a rat model of MIRI or ICons. As a technical limitation, we can disclose that MMP activities measured by gelatin zymography might be overinterpreted since zymography is performed in an artificial environment lacking the endogenous tissue inhibitors of MMPs (TIMPs) or the circulatory alpha-2-macroglobuline, and, therefore, the method is not able to measure actual in vivo activity; instead, activity potential can only be determined (see also at [13]).

## 6. Conclusions

Our present original work was on myocardial ischemia/reperfusion injury as well as endogenous cardioprotective adaptive mechanisms, i.e., ischemic conditionings by using small and large animal models with a high translational value to human disease. We have investigated changes in myocardial microRNA patterns with a particular focus on microRNAs involved in oxidative stress and related to matrix metalloproteinases and biglycan signaling mechanisms. In this way, a complex approach based on experimental measurements, available databases, and bioinformatic analysis has provided valuable insights into the network of myocardial miRNAs and MMP—biglycan and/or oxidative stress/antioxidant signaling pathways, thereby establishing future studies and potential novel drug developments against myocardial ischemia/reperfusion injury.

## Figures and Tables

**Table 1:** List of predicted regulatory miRNAs and their measured porcine homologous miRNAs with significantly altered expression (*p* < 0.05; absolute value of fold change > 1.5) in order of the magnitude of expression change due to ischemic injury. Empty cells indicate non-significant changes. Node degree shows the number of targets with which the human miRNA is predicted to interact.

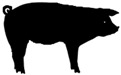	Human miRNA Name	Target Pathway	Node Degree	Sham vs. I/R Log2 Fold Change	I/R vs. IPreC Log2 Fold Change	I/R vs. IPostC Log2 Fold Change	I/R vs. RIPerC Log2 Fold Change
Porcine miRNA Name							
miR-369	hsa-miR-369-3p	MMP	1	2.55		−2.55	−1.78
ssc-miR-34a	hsa-miR-34a-5p	BGN, MMP	2; 10	2.20	−0.91	−0.68	−0.83
miR-196a	hsa-miR-196a-5p	MMP	3	1.78		−1.53	−1.45
ssc-miR-128	hsa-miR-128-3p	BGN, MMP	2; 7	1.78			−16.02
ssc-miR-9-2	hsa-miR-9-5p	BGN, MMP	3; 14	1.74			−0.88
ssc-miR-23a	hsa-miR-23a-3p	MMP	3	1.17		−0.94	−15.81
ssc-miR-126	hsa-miR-126-3p	MMP	3	1.39			1.04
ssc-miR-101a	hsa-miR-101-3p	MMP	5	1.18	1.24		
ssc-miR-193a-3p	hsa-miR-193a-3p	BGN, MMP	1; 1	1.05	−11.21	−1.36	−1.44
ssc-miR-193a-5p	hsa-miR-193a-5p	MMP	1	0.93		−1.05	−0.69
ssc-miR-338	hsa-miR-338-3p	MMP	5	0.60		−1.07	
ssc-miR-320c	hsa-miR-320c	MMP	1	−10.50	12.51		
ssc-miR-7-1	hsa-miR-7-5p	MMP	5	−8.93	−8.71		−13.61
ssc-miR-106b	hsa-miR-106b-5p	BGN, MMP	4; 10	−4.06			
ssc-miR-18b	hsa-miR-18b-5p	BGN, MMP	2; 1	−2.72		−8.18	−4.54
ssc-miR-30b-5p	hsa-miR-30b-5p	BGN, MMP	1; 3	−2.56	−1.33		−5.53
ssc-miR-215	hsa-miR-215-5p	MMP	3	−2.44			0.71
ssc-miR-361-5p	hsa-miR-361-5p	BGN, MMP	1; 1	−1.88	1.48	1.77	
ssc-miR-450a	hsa-miR-450a-5p	MMP	1	−1.41	1.88	2.00	2.74
ssc-miR-107	hsa-miR-107	BGN, MMP	1; 2	−1.27	0.73		0.73
ssc-miR-26a	hsa-miR-26a-5p	BGN, MMP	4; 3	−1.24	0.77		1
ssc-miR-135	hsa-miR-135a-5p	MMP	3	−1.08			
let-7a	hsa-let-7a-5p	BGN, MMP	2; 4	−0.79			
ssc-miR-19b	hsa-miR-19b-3p	BGN, MMP	4; 4	−0.75			
ssc-miR-425-5p	hsa-miR-425-5p	BGN, MMP	1; 1	−0.68			0.72
ssc-let-7e	hsa-let-7e-5p	BGN, MMP	1; 3			3.51	3.75
ssc-miR-15b	hsa-miR-15b-5p	BGN, MMP	2; 3		12.14		8.23
ssc-miR-130a	hsa-miR-130a-3p	BGN, MMP	3; 6		−0.65	−0.95	
ssc-miR-130b	hsa-miR-130b-3p	BGN, MMP	1; 7			−2.45	
ssc-miR-145-3p	hsa-miR-145-3p	MMP	1				0.70
ssc-miR-151-5p	hsa-miR-151a-5p	MMP	1		0.79		0.82
ssc-miR-185	hsa-miR-185-5p	BGN, MMP	1; 2			4.43	
ssc-miR-204a	hsa-miR-204-5p	BGN, MMP	3; 7		−0.90	−0.86	−0.77
ssc-miR-211	hsa-miR-211-5p	BGN, MMP	1; 3			−2.22	
ssc-miR-365-3p	hsa-miR-365a-3p	BGN, MMP	1; 1		0.80		
ssc-miR-374b	hsa-miR-374b-5p	MMP	2		0.62		
ssc-miR-455-3p	hsa-miR-455-3p	MMP	1				1.17

**Table 2:** List of predicted regulatory miRNAs and their measured rat homologous miRNAs with significantly altered expression (*p* < 0.05; absolute value of fold change >1.5) in order of the magnitude of expression change due to ischemic injury. Empty cells indicate non-significant changes. Node degree shows the number of targets with which the human miRNA is predicted to interact.

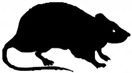	HumanmiRNA Name	Target Pathway	NodeDegree	Sham vs. I/R Log2 Fold Change	I/R vs. IPreC Log2 Fold Change	I/R vs. IPostC Log2 Fold Change
Rat miRNA Name						
rno-miR-19b-3p	hsa-miR-19b-3p	BGN, MMP	4; 4	0.70		
rno-miR-19a-3p	hsa-miR-19a-3p	BGN, MMP	3; 2	0.60		
rno-miR-33-5p	hsa-miR-33a-5p	BGN, MMP	1; 4	−1.61		
rno-let-7b-5p	hsa-let-7b-5p	BGN, MMP	2; 7	−1.07		1.09
rno-miR-335	hsa-miR-335-5p	BGN, MMP	5; 14	−0.99	−0.66	1.37
rno-miR-320-3p	hsa-miR-320a	MMP	7	−0.95	0.75	0.74
rno-miR-331-3p	hsa-miR-331-3p	MMP	5	−0.92		
rno-let-7c-5p	hsa-let-7c-5p	BGN, MMP	2; 4	−0.92		0.94
rno-let-7a-5p	hsa-let-7a-5p	BGN, MMP	2; 4	−0.84		0.79
rno-miR-125a-5p	hsa-miR-125a-5p	BGN, MMP	3; 3	−0.82		
rno-miR-378a-5p	hsa-miR-378a-5p	MMP	2	−0.81		
rno-miR-652-3p	hsa-miR-652-3p	BGN	1	−0.75		
rno-miR-181a-5p	hsa-miR-181a-5p	BGN, MMP	2; 8	−0.71		0.62
rno-miR-877	hsa-miR-877-5p	MMP	3	−0.76		
rno-miR-218a-5p	hsa-miR-218-5p	BGN, MMP	1; 10	−0.67		
rno-let-7d-5p	hsa-let-7d-5p	BGN, MMP	1; 1	−0.66		0.71
rno-let-7f-5p	hsa-let-7f-5p	BGN, MMP	2; 2	−0.62		0.66
rno-let-7i-5p	hsa-let-7i-5p	BGN	1			0.68
rno-let-7e-5p	hsa-let-7e-5p	BGN, MMP	1; 3			0.80
rno-miR-93-5p	hsa-miR-93-5p	BGN, MMP	5; 11			−0.91
rno-miR-188-5p	hsa-miR-188-5p	BGN	1		1.08	0.69
rno-miR-192-5p	hsa-miR-192-5p	MMP	3		0.92	
rno-miR-532-3p	hsa-miR-532-3p	BGN, MMP	1; 2		1.07	1.09
rno-miR-874-3p	hsa-miR-874-3p	MMP	2		1.06	0.88

**Table 3:** Differentially expressed miRNAs (*p* < 0.05; absolute value of fold change >1.5) which are counter-regulated by ischemic conditioning protocols in relation to MMP-2, -9, and/or BGN regulation. Empty cells indicate non-significant changes. Node degree shows the number of targets with which the human miRNA is predicted to interact. I/R: ischemia/reperfusion injury only, IPreC: ischemic preconditioning, IPostC: ischemic postconditioning, RIPerC remote ischemic perconditioning on hind limb, MMP: matrix metalloproteinase, BGN: biglycan.

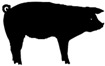	HumanmiRNA Name	Target Pathway	Node Degree	Sham vs. I/R Log2 Fold Change	I/R vs. IPreC Log2 Fold Change	I/R vs. IPostC Log2 Fold Change	I/R vs. RIPerC Log2 Fold Change	Direction of Expression Change after ICons
miRNA Name								
ssc-miR-34a	hsa-miR-34a-5p	BGN, MMP	2; 10	2.20	−0.91	−0.68	−0.83	Downregulation
ssc-miR-193a-3p	hsa-miR-193a-3p	BGN, MMP	1; 1	1.05	−11.21	−1.36	−1.44	
ssc-miR-193a-5p	hsa-miR-193a-5p	MMP	1	0.93		−1.05	−0.69	
ssc-miR-23a	hsa-miR-23a-3p	MMP	3	1.17		−0.94	−15.81	
miR-196a	hsa-miR-196a-5p	MMP	3	1.78		−1.53	−1.45	
miR-369	hsa-miR-369-3p	MMP	1	2.55		−2.55	−1.78	
ssc-miR 9-2	hsa-miR-9-5p	BGN, MMP	3; 14	1.74			−0.88	
ssc-miR-128	hsa-miR-128-3p	BGN, MMP	2; 7	1.78			−16.02	
ssc-miR-338	hsa-miR-338-3p	MMP	5	0.60		−1.07		
ssc-miR-450a	hsa-miR-450a-5p	MMP	1	−1.41	1.88	2.00	2.74	Upregulation
ssc-miR-26a	hsa-miR-26a-5p	BGN, MMP	4; 3	−1.24	0.77		1	
ssc-miR-107	hsa-miR-107	BGN, MMP	1; 2	−1.27	0.73		0.73	
ssc-miR-361-5p	hsa-miR-361-5p	BGN, MMP	1; 1	−1.88	1.48	1.77		
ssc-miR-215	hsa-miR-215-5p	MMP	3	−2.44			0.71	
ssc-miR-425-5p	hsa-miR-425-5p	BGN, MMP	1; 1	−0.68			0.72	
ssc-miR-320c	hsa-miR-320c	MMP	1	−10.50	12.51			
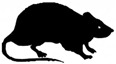								
miRNA Name								
rno-miR-320-3p	hsa-miR-320a	MMP	7	−0.95	0.75	0.74		Upregulation
rno-let-7a-5p	hsa-let-7a-5p	BGN, MMP	2; 4	−0.84		0.79		
rno-let-7b-5p	hsa-let-7b-5p	BGN, MMP	2; 7	−1.07		1.09		
rno-let-7c-5p	hsa-let-7c-5p	BGN, MMP	2; 4	−0.92		0.94		
rno-let-7d-5p	hsa-let-7d-5p	BGN, MMP	1; 1	−0.66		0.71		
rno-let-7f-5p	hsa-let-7f-5p	BGN, MMP	2; 2	−0.62		0.66		
rno-miR-181a-5p	hsa-miR-181a-5p	BGN, MMP	2; 8	−0.71		0.62		
rno-miR-335	hsa-miR-335-5p	BGN, MMP	5; 14	−0.99		1.37		

**Table 4:** Relationship of differentially expressed miRNAs involved in the regulation of MMP-2, -9, and/or BGN, and that are counter-regulated by ischemic conditionings, to cardiac oxidative stress or to the antioxidant protection of cardiac myocytes. In “All hits” column, the number of PubMed hits is indicated according to the search strings “miR-NN AND (“oxidative stress” OR “nitrative stress” OR “nitrosative stress” OR antioxidant OR “free radical”)”.

miRNA	Cardiac Oxidative Stress-Related Results	Model	Reference	All Hits
miR-9-5p	MiR-9-5p expression was associated with cellular injury. Transfection of miR-9-5p mimics in hypoxic H9c2 cells increased cardiac cell death and increased ROS formation as shown by elevated malondialdehyde levels. Moreover, silencing of miR-9-5p by a specific antagomir in vivo in a mouse acute MI model, effectively preserved post-MI heart function with attenuated fibrosis and inflammatory responses.	H9c2 cells, primary culture of neonatal rat ventricular myocytes male C57BL/6 mice	[37]	56
miR-23a	Doxorubicin treatment reduced cell viability and increased ROS production and miR-23a expression, which was reversible with miR-23a inhibitor.	Neonatal rat cardiomyocyte	[38]	43
miR-26a-5p	Lipopolysaccharide-induced apoptosis in HL-1 cells and in mice was reversible with miR-26a-5p agomiR treatment through decreased ROS formation, as shown by decreased malondialdehyde and increased anti-oxidant levels measured as glutathione and glutathione peroxidase.	Lipopolysaccharide-induced sepsis in HL-1 cells and C57BL/6J mice	[39]	45
miR-34a-5p	Intracardiac administration of miR-34a-5p antagomir decreased apoptosis and fibrosis in myocardial tissue at 4 weeks following coronary ligation.	male C57BL/6J mice; coronary artery ligation with 4-week follow-up	[40]	227
miR-107	Overexpression of miR-103/107 resulted in a significant increase in cell necrosis after high dose H_2_O_2_ exposure in H9c2 cells, which was mediated by the reduction of Fas-associated protein with death domain (FADD) protein. MIRI led to a reduction in FADD, and that reduction was attenuated by miR-103/107 knockdown. The administration of miR-103/107 antagomir resulted in a reduction in myocardial necrosis in infarct size as well as in troponin T levels.	H_2_O_2_-induced necrotic cell death in H9c2 cells; male C57BL/6J mice subjected to myocardial ischemia/reperfusion injury (MIRI)	[41]	17
miR-128	Homocysteine treatment reduced cell viability and superoxide dismutase activity and increased malondialdehyde and ROS production, which was reversible with miR-128 mimic treatment.	Primary rat cardiac microvascular endothelial cells (CMECs); homocysteine-induced cell injury	[42]	32
miR-181a-5p	Fetal and neonatal nicotine exposure-mediated miR-181a overexpression plays an important role in nicotine-enhanced coronary vascular tone via epigenetic downregulation of BKca channel mechanism, which provides a potentially novel therapeutic molecular target of miR-181a/BKca channels for the treatment of coronary heart disease.	isolated coronary arteries, isolated coronary arterial smooth muscle cells (SMCs)	[43]	65
	Cardiac specific miR-181a transgenic mice showed a significant decrease in the amount of ischemia-induced apoptosis as well as myocardial infarct size 24 h after induction of MI as measured by TUNEL and by TTC staining, respectively. Furthermore, cardiac specific overexpression of miR-181a preserved left ventricular function after MI. Cardioprotection by miR-181a was associated with programmed cell death protein 4 (PDCD4) as a target gene.	Primary cultures of neonatal rat cardiac ventricular myocytes cardiac-specific miR-181a transgenic and their lean mice	[44]	
miR-193a-5p	Administration of miR-193a-5p to HUVEC cell cultures directly protected cells from H_2_O_2_-induced oxidative damage. Isolated circulating exosomes from AMI patients (AMI-Exo) showed decreased expression of miR-193a-5p. In a rat carotid artery balloon injury model, miR-193a-5p mimic was transfected into AMI-Exo through electroporation, where quantitative analysis of intima/media ratios showed that miR-193a-5p was essential to protect endothelial cells.	Human umbilical vein endothelial cells’ (HUVECs) cell culture; isolated exosomes from venous whole blood samples of AMI patients; rat carotid artery balloon injury model	[45]	17
miR-215-5p	Increased ROS production was shown in chicken cardiomyocytes treated with miR-215-5p mimic.	Isolated chicken cardiomyocyte	[46]	11
miR-320	In vitro hypoxia-induced apoptosis was reversible with antagomir-320 treatment. In vivo ischemia/reperfusion induced injury increased malondialdehyde and decreased superoxide dismutase content, which effect was reversible with antagomiR-320 treatment.	Ischemia/reperfusion injury in Sprague–Dawley rat, simulated ischemic injury in H9c2-cells.	[47]	18
miR-335-5p	Overexpression of mir-335 by intravenous administration of agomiR-335 decreased myocardial infarct size and improved cardiac function at least partially via decreasing oxidative stress as characterized by increased SOD and decreased malondialdehyde levels as compared to the negative control-treated infarcted rats.	male Sprague–Dawley rats subjected to MIRI	[48]	22
miR-361-5p	Lipopolysaccharide-induced sepsis increased cardiomyocyte apoptosis and upregulated miR-361-5p expression, which was reversible with knockdown of miR-361-5p.	Lipopolysaccharide-induced myocardial injury in mouse	[49]	8
miR-425-5p	Doxorubicin-induced cardiac dysfunction and cytotoxicity resulted in miR-425 downregulation in vitro and in vivo. MiR-425 mimic treatment reduced doxorubicin-induced ROS and apoptosis in vitro.	male C57BL/6 mice; HL-1—mouse cardiac muscle cell line	[50]	8

## Data Availability

The data that presented in this study are available from the corresponding author upon reasonable request.

## Supplementary Materials

The following supporting information can be downloaded at https://www.mdpi.com/article/10.3390/antiox13060674/s1, Figure S1: Representative gelatin zymogram from porcine ischemic left ventricular samples; Figure S2: Interaction network of *biglycan* regulatory genes and predicted interacting human miRNAs; Figure S3: Interaction network of *matrix metalloproteinase* regulatory genes and predicted interacting human miRNAs; Table S1: Genes used as input for network theoretical reverse target-miRNA prediction; Table S2: Significantly changed *porcine* cardiac miRNAs that were predicted to interact with targets used as input for the network theoretical reverse target-miRNA prediction; Table S3: Significantly changed *rat* cardiac miRNAs that were predicted to interact with targets used as input for the network theoretical reverse target-miRNA prediction.
