# Supplementary material for: Identification of New, Translatable ProtectomiRs against Myocardial Ischemia/Reperfusion Injury and Oxidative Stress: The Role of MMP/Biglycan Signaling Pathways
Source: Antioxidants (Basel). 2024 May 30;13(6):674. doi: 10.3390/antiox13060674 (PMC11201193; doi:10.3390/antiox13060674)
Supplement: Supplementary file 1 [file antioxidants-13-00674-s001.zip › antioxidants-2979880-supplementary.pdf]

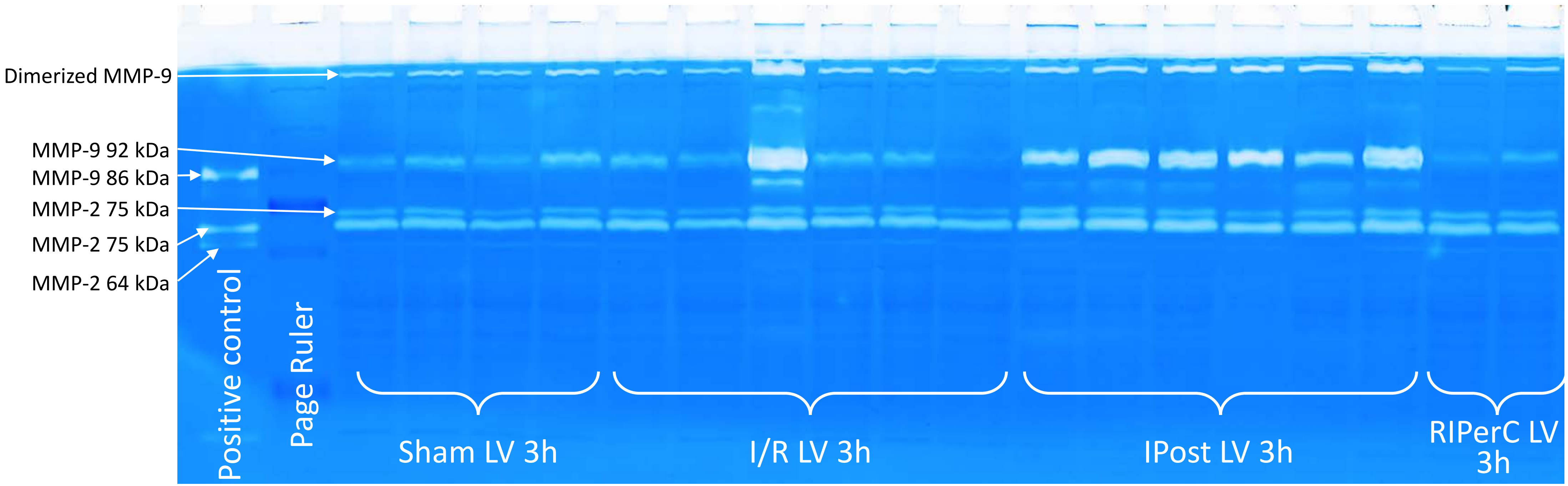

Supplementary Figure S1. Representative gelatin zymogram from porcine ischemic left ventricular samples. I/R, ischemia/reperfusion; IPost, ischemic postconditioned group; RIPerC, remote ischemic perconditioned group.

Biglycan interacting regulatory targets	MMP-2 and -9 interacting regulatory targets		
BGN	MMP2	HTATIP2	SNAI2
TGFB1	MMP9	IKBKB	SP1
TGFB2	A2M	IRF1	SP2
TGFB3	TIMP2	JUN	SP3
TGFB4	TIMP4	KLF4	SPDEF
SMAD2	PLG	KLF5	SRF
SMAD4	F2	KLF6	SSX2
MAPK14	ATF2	KLF8	STAT1
PRKCA	ATF3	MAZ	STAT3
PRKCB	CEBPE	MSC	TFAP2A
PRKCG	CIITA	MTA1	TFAP2C
CREB1	CREB1	MZF1	TFCP2
F2	ELF3	NFKB1	TP53
TNF	ELF4	NFKBIA	TWIST1
PDGFB	EP300	NR4A1	TWIST2
IL1A	ETS1	PAX6	YBX1
IL1B	ETS2	PPARA	
IL6	ETV4	PPARG	
AGT	EZH2	PTTG1	
MSTN	FOS	RELA	
NOS1	HDAC1	RUNX2	
NOS2	HDAC3	RUNX3	
NOS3	HIF1A	SIRT1	
CTGF	HOXB7	SMAD3	

Supplementary Table S1. Genes used as input for network theoretical reverse target-miRNA prediction.

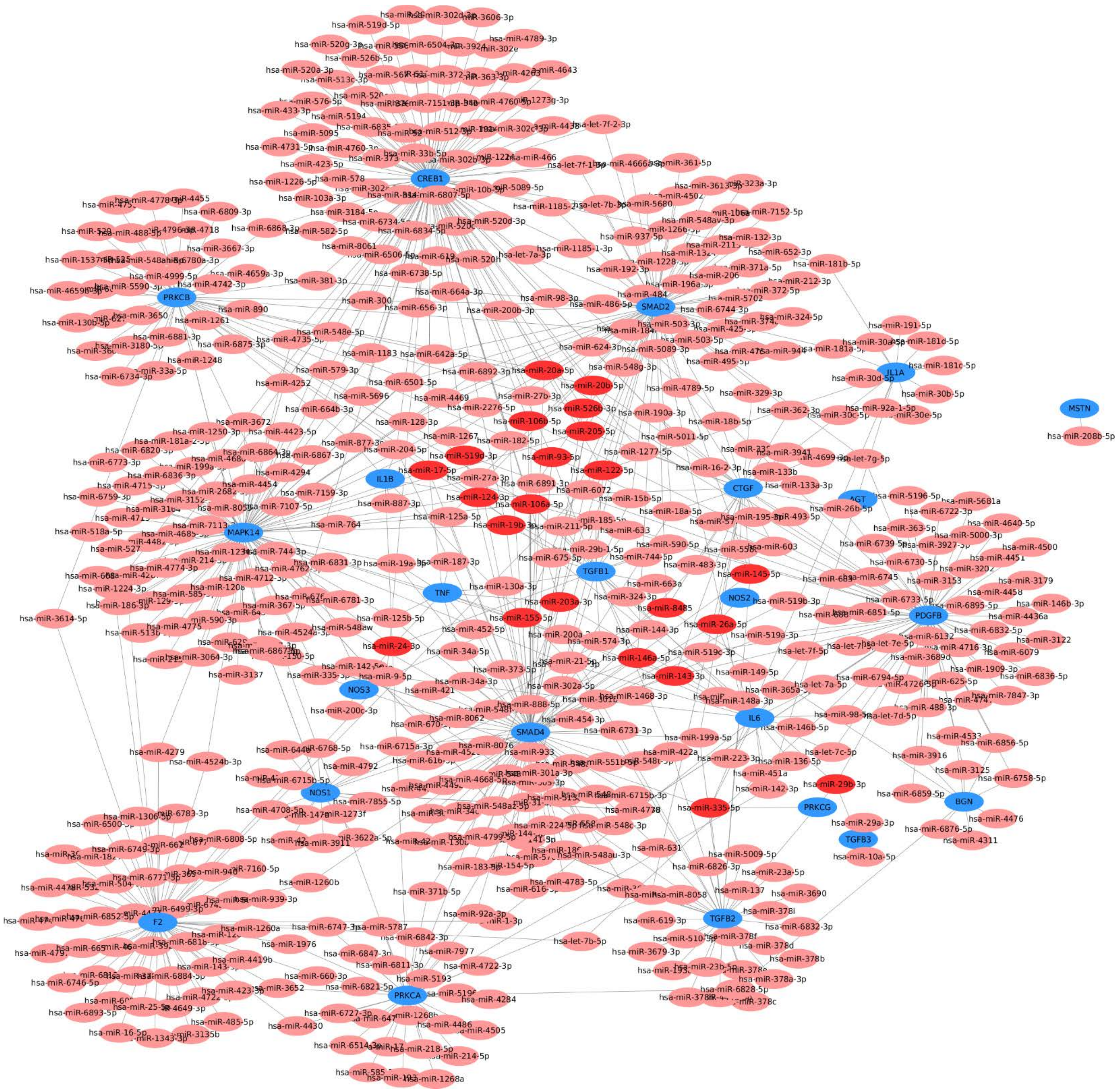

Supplementary Figure S2. Interaction network of ***biglycan*** regulatory genes and predicted interacting human miRNAs. Genes are indicated in light-blue colour, miRNAs with 2 or 3 interacting genes are indicated in orange colour, and at least four interacting genes are indicated in dark-red colour.

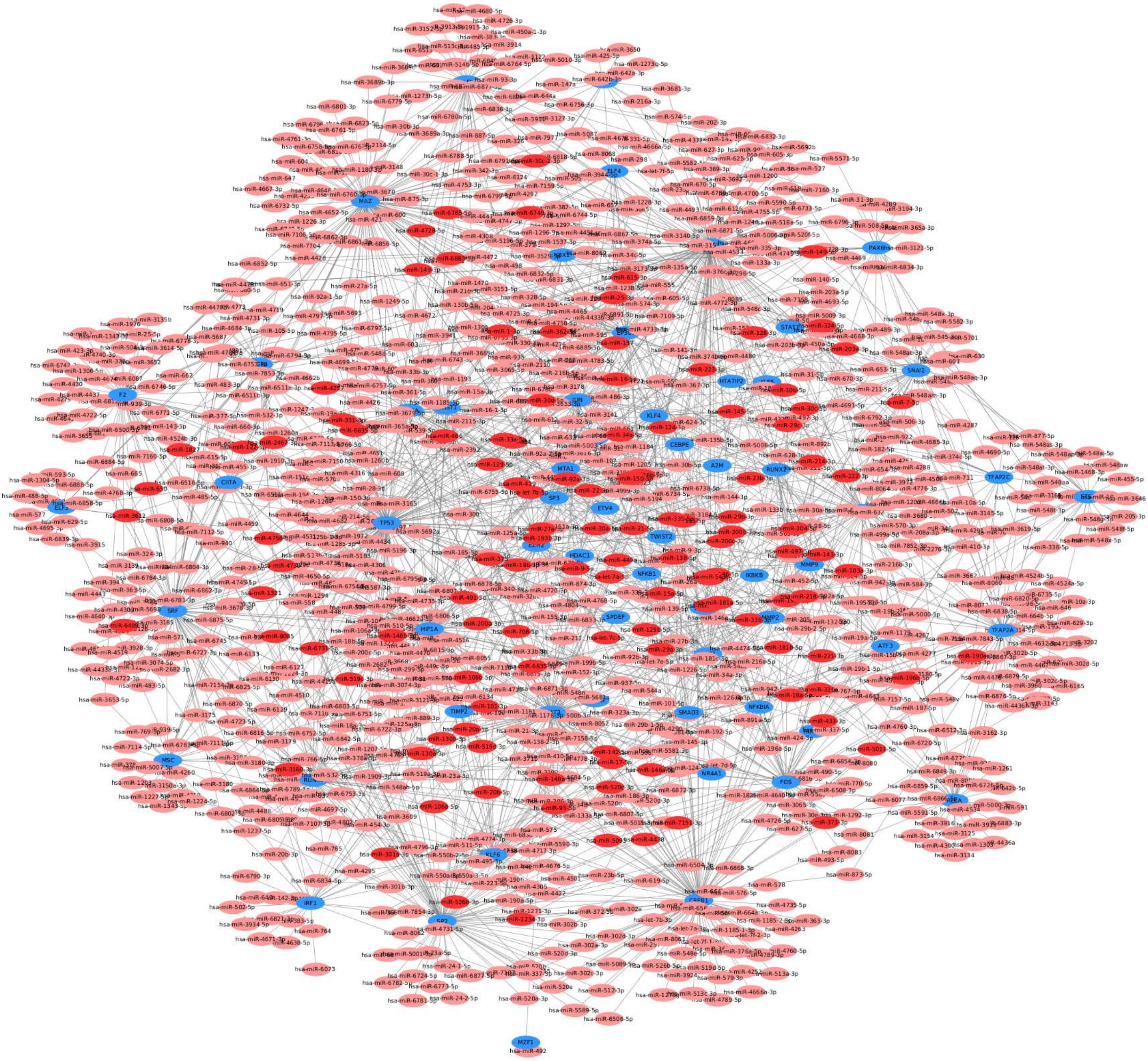

Supplementary Figure S3. Interaction network of ***matrix metalloproteinase*** regulatory genes and predicted interacting human miRNAs. Genes are indicated in light-blue colour, miRNAs with two or three interacting genes are indicated in orange colour, and at least four interacting genes are indicated in dark-red colour.

Porcine miRNA Name	Human miRNA Name	Interacting targets in MMP-2 and -9, and/or biglycan regulatory pathway
ssc-miR-9-2	hsa-miR-9-5p	KLF6;CREB1;ELF3;EP300;ETS1;F2;SIRT1;MMP2;MMP9;NFKB1;PPARA;SNAI2;SRF;KLF5;F2;IL6
ssc-miR-106b	hsa-miR-106b-5p	KLF6;CREB1;HIF1A;MMP2;YBX1;SP2;STAT3;TP53;TWIST1;RUNX3;SMAD4;PDGFB;PRKCB
ssc-miR-34a	hsa-miR-34a-5p	SIRT1;FOS;HDAC1;MAZ;NFKB1;YBX1;PPARA;STAT1;TP53;KLF4;SMAD4;TNF
ssc-miR-128	hsa-miR-128-3p	A2M;SIRT1;SNAI2;SP1;SSX2;KLF5;KLF4;MAPK14;SMAD2
ssc-miR-130a	hsa-miR-130a-3p	KLF6;IRF1;PPARA;PPARG;RUNX3;KLF4;SMAD4;TGFB1;TNF
ssc-miR-204a	hsa-miR-204-5p	CREB1;SIRT1;SPDEF;MMP9;SNAI2;SP1;RUNX2;IL1B;SMAD4
ssc-miR-130b	hsa-miR-130b-3p	KLF6;IRF1;MMP2;PPARA;PPARG;STAT3;RUNX3;SMAD4
ssc-miR-19b	hsa-miR-19b-3p	CREB1;ATF2;PPARA;TP53;MAPK14;SMAD4;TGFB1
miR-26a	hsa-miR-26a-5p	EP300;EZH2;PTTG1;CTGF;IL6;SMAD4;NOS2
let-7a	hsa-let-7a-5p	EZH2;NFKB1;SP1;STAT3;IL6;PDGFB
ssc-miR-101a	hsa-miR-101-3p	KLF6;EZH2;FOS;JUN;SRF
ssc-miR-15b	hsa-miR-15b-5p	TGFB1;SMAD2;SMAD3;MMP9;TFAP2A
ssc-miR-338	hsa-miR-338-3p	FOS;HIF1A;MMP2;MMP9;RUNX2
ssc-miR-7-1	hsa-miR-7-5p	FOS;MAZ;PAX6;RELA;KLF4
ssc-let-7e	hsa-let-7e-5p	EZH2;MMP9;STAT3;PDGFB
ssc-miR-211	hsa-miR-211-5p	MMP9;SNAI2;SP1;TGFB1
ssc-miR-30b-5p	hsa-miR-30b-5p	TP53;RUNX2;KLF4;IL1A
ssc-miR-107	hsa-miR-107	HIF1A;KLF4;IL6
ssc-miR-126	hsa-miR-126-3p	EZH2;SIRT1;NFKBIA
ssc-miR-135	hsa-miR-135a-5p	KLF8;RUNX2;KLF4
ssc-miR-185	hsa-miR-185-5p	EZH2;TP53;TGFB1
ssc-miR-18b	hsa-miR-18b-5p	HIF1A;CTGF;SMAD2
miR-196a	hsa-miR-196a-5p	NR4A1;HOXB7;NFKBIA
ssc-miR-215	hsa-miR-215-5p	SIRT1;FOS;PPARG
ssc-miR-23a	hsa-miR-23a-3p	IRF1;SMAD3;STAT3
ssc-miR-193a-3p	hsa-miR-193a-3p	IRF1;TGFB2
ssc-miR-361-5p	hsa-miR-361-5p	TWIST1;SMAD2
ssc-miR-365-3p	hsa-miR-365a-3p	PAX6;IL6
ssc-miR-374b	hsa-miR-374b-5p	ATF2;SP1
ssc-miR-425-5p	hsa-miR-425-5p	SSX2;SMAD2
ssc-miR-145-3p	hsa-miR-145-3p	SMAD3
ssc-miR-151-5p	hsa-miR-151a-5p	TP53
ssc-miR-193a-5p	hsa-miR-193a-5p	TFAP2A
ssc-miR-320c	hsa-miR-320c	EZH2
miR-369	hsa-miR-369-3p	SP1
ssc-miR-450a	hsa-miR-450a-5p	STAT1
ssc-miR-455-3p	hsa-miR-455-3p	CIITA

Supplementary Table S2. Significantly changed ***porcine*** cardiac miRNAs that were predicted to interact with targets used as input for the network theoretical reverse target-miRNA prediction.

Rat miRNA Name	Human miRNA Name	Interacting targets in MMP-2 and -9, and/or biglycan regulatory pathway
rno-miR-335	hsa-miR-335-5p	KLF8;ATF2;FOS;SPDEF;NR4A1;IKBKB;SMAD3;CIITA;MMP2;NFKBIA;PPARA;SP1;RUNX2;KLF4;IL1A;IL6;NOS3;PRKCG;TGFB2
rno-miR-93-5p	hsa-miR-93-5p	KLF6;CREB1;EZH2;HIF1A;NR4A1;IRF1;JUN;NFKBIA;SP2;STAT3;RUNX3;SMAD4;PDGFB;PRKCB;TGFB1
rno-miR-218a-5p	hsa-miR-218-5p	ETS2;IKBKB;MMP2;PPARG;SNAI2;SP1;SP3;RUNX2;RUNX3;MSC;PRKCA
rno-miR-181a-5p	hsa-miR-181a-5p	KLF6;ETS1;SIRT1;FOS;MAZ;SNAI2;STAT3;TWIST1;IL1A;SMAD2
rno-let-7b-5p	hsa-let-7b-5p	EP300;EZH2;F2;HIF1A;NFKBIA;SP1;PTTG1;PDGFB
rno-miR-320-3p	hsa-miR-320a	EZH2;MMP9;RELA;SRF;TFAP2A;RUNX2;PTTG1
rno-miR-19b-3p	hsa-miR-19b-3p	CREB1;ATF2;PPARA;TP53;MAPK14;SMAD4;TGFB1
rno-let-7a-5p	hsa-let-7a-5p	EZH2;NFKB1;SP1;STAT3;IL6;PDGFB
rno-let-7c-5p	hsa-let-7c-5p	EZH2;SIRT1;SPDEF;STAT3;IL6;PDGFB
rno-miR-331-3p	hsa-miR-331-3p	SIRT1;MAZ;ATF3;SRF;TFCP2
rno-miR-33-5p	hsa-miR-33a-5p	HIF1A;PPARA;SP1;TWIST1;PRKCB
rno-miR-125a-3p	hsa-miR-125a-3p	EZH2;IRF1;MTA1;MAPK14;IL6
rno-miR-19a-3p	hsa-miR-19a-3p	PPARA;TP5;MAPK14;SMAD4;TNF
rno-let-7e-5p	hsa-let-7e-5p	EZH2;MMP9;STAT3;PDGFB
rno-let-7f-5p	hsa-let-7f-5p	ELF4;SP1;IL6;PDGFB
rno-miR-192-5p	hsa-miR-192-5p	SIRT1;FOS;PPARG
rno-miR-877	hsa-miR-877-5p	ELF4;ETS2;RELA
rno-miR-378a-5p	hsa-miR-378a-5p	IRF1;SP1
rno-miR-532-3p	hsa-miR-532-3p	EZH2;F2
rno-miR-874-3p	hsa-miR-874-3p	HDAC1;STAT3
rno-let-7d-5p	hsa-let-7d-5p	NR4A1;PDGFB
rno-let-7i-5p	hsa-let-7i-5p	PDGFB
rno-miR-188-5p	hsa-miR-188-5p	SMAD2
rno-miR-652-3p	hsa-miR-652-3p	SMAD2

Supplementary Table S3. Significantly changed ***rat*** cardiac miRNAs that were predicted to interact with targets used as input for the network theoretical reverse target-miRNA prediction.
